# Surgical Technique and Fertility Outcomes: A Comprehensive Review of Open and Laparoscopic Cystectomy in Women of Reproductive Age

**DOI:** 10.7759/cureus.71179

**Published:** 2024-10-10

**Authors:** Neha Sethi, Manjusha Agrawal, Archan Patel, Lucky Srivani Reddy, Drishti M Bhatt

**Affiliations:** 1 Obstetrics and Gynecology, Jawaharlal Nehru Medical College, Datta Meghe Institute of Higher Education & Research, Wardha, IND; 2 Dermatology, Jawaharlal Nehru Medical College, Datta Meghe Institute of Higher Education & Research, Wardha, IND

**Keywords:** cystectomy, fertility, laparoscopic surgery, open surgery, reproductive health, surgical technique

## Abstract

Cystectomy, the surgical removal of ovarian tissue, is commonly performed in women of reproductive age to address conditions such as ovarian cysts, endometriosis, and tumors. The choice of surgical technique, open versus laparoscopic, has significant implications for postoperative recovery and long-term fertility outcomes. This comprehensive review aims to evaluate the current literature on the effects of these two surgical approaches on fertility in women of reproductive age. Open cystectomy, while effective, is associated with larger incisions, increased trauma to surrounding reproductive structures, and a higher incidence of postoperative complications, which may negatively impact future fertility. In contrast, laparoscopic cystectomy offers a minimally invasive option that generally results in less postoperative pain, quicker recovery, and potentially improved fertility outcomes due to reduced damage to surrounding tissues. However, the literature reveals a complex interplay between surgical technique, underlying medical conditions, and individual patient factors that can influence reproductive potential. This review synthesizes key studies comparing the fertility outcomes of both surgical methods, highlighting the need for individualized surgical planning based on each patient's unique circumstances and reproductive goals. Additionally, it discusses the importance of preoperative counseling and multidisciplinary approaches to optimize patient outcomes. Future research is essential to further clarify the long-term fertility implications of open and laparoscopic cystectomy and to refine surgical techniques to enhance reproductive health. This review contributes to the growing body of evidence guiding clinicians in making informed decisions that prioritize the effective treatment of ovarian pathology and fertility preservation.

## Introduction and background

Cystectomy, the surgical removal of one or both ovaries and any associated cysts, is a prevalent procedure among women of reproductive age. This surgical intervention is often indicated for various ovarian conditions, including benign ovarian cysts, endometriosis, and malignant tumors [1]. Ovarian cysts, which can lead to pain, hormonal imbalances, and reproductive issues, may necessitate surgical intervention to alleviate symptoms and prevent complications. For women facing the prospect of cystectomy, the choice of surgical technique, open versus laparoscopic, can significantly influence both immediate postoperative recovery and long-term reproductive health [2]. As the demand for effective management of ovarian conditions continues to rise, understanding the implications of cystectomy becomes increasingly crucial for optimizing patient outcomes [2].

The importance of surgical technique in cystectomy cannot be overstated, especially concerning fertility outcomes. Open cystectomy involves a larger incision and a more invasive approach, which can result in greater tissue trauma, increased postoperative pain, and longer recovery times [3]. This technique may also pose a higher risk of complications, such as infection and adhesion formation, which could adversely affect future fertility. Conversely, laparoscopic cystectomy, characterized by smaller incisions and a minimally invasive approach, has gained popularity due to its potential advantages, including reduced pain, quicker recovery, and less damage to surrounding reproductive structures. The differences in these techniques warrant a thorough examination, as they may influence a woman's ability to conceive in the future [3].

This review aims to comprehensively evaluate the current literature surrounding the surgical techniques of open and laparoscopic cystectomy, specifically focusing on their respective impacts on fertility outcomes in women of reproductive age. By synthesizing findings from clinical studies, meta-analyses, and expert opinions, this review seeks to provide valuable insights for healthcare providers and patients alike. The ultimate objective is to enhance understanding of how surgical decisions can be tailored to preserve fertility while effectively addressing ovarian pathology. Through this exploration, we hope to contribute to the existing body of knowledge and assist clinicians in making informed decisions that prioritize both the immediate and long-term reproductive health of women undergoing cystectomy.

## Review

Overview of cystectomy

Cystectomy is a surgical procedure that involves the removal of all or part of the bladder. While it is primarily performed to treat bladder cancer, it may also be indicated for other serious bladder conditions. Cystectomies are categorized into two main types based on the extent of bladder removal: radical cystectomy and partial cystectomy [3]. A radical cystectomy entails the complete removal of the bladder, along with surrounding lymph nodes and potentially other pelvic organs. In women, this may include the uterus, cervix, fallopian tubes, and ovaries; in men, it may involve the prostate and seminal vesicles. Conversely, a partial cystectomy, also known as a segmental cystectomy, involves the removal of only a portion of the bladder affected by disease, typically cancer. This approach allows for preserving bladder function, although it may reduce capacity [4]. Cystectomies can be performed using two surgical techniques: open cystectomy and laparoscopic cystectomy. Open cystectomy involves a larger abdominal incision to access the bladder directly. In contrast, laparoscopic cystectomy is a minimally invasive technique that employs small incisions and specialized instruments, often assisted by robotic technology. The laparoscopic approach generally leads to quicker recovery times and less postoperative pain compared to open surgery [5].

The indications for cystectomy in women of reproductive age primarily center around bladder cancer, which is the most common reason for this procedure. When cancer has invaded the muscle layer of the bladder, a cystectomy may be necessary to ensure the complete removal of malignant tissue. Additionally, benign conditions such as severe interstitial cystitis or congenital anomalies affecting bladder function may also warrant this surgery. Patients with recurrent or treatment-resistant conditions may be recommended for cystectomy when other treatments have failed or when there is a high risk of cancer recurrence [6]. While cystectomy offers significant benefits, it is not without risks. The primary advantage of this procedure is its effectiveness in treating cancerous tissue, which can greatly improve prognosis and quality of life for patients. In benign conditions, cystectomy can alleviate symptoms such as pain or urinary dysfunction. Moreover, successful treatment can improve urinary function post-surgery, particularly with partial cystectomies or effective reconstructive techniques. However, patients must also consider the associated risks [7]. As with any major surgery, there are potential risks of bleeding, infection, and complications related to anesthesia. Patients may experience changes in urinary function after surgery, such as increased frequency or issues related to urinary diversion methods. Additionally, there is a risk of sexual dysfunction due to nerve damage during surgery, which can affect both men and women differently, depending on the extent of the surgery performed [8].

Surgical techniques

Open cystectomy, also known as laparotomy, is a surgical procedure that involves the removal of an ovarian cyst through a large abdominal incision. This technique is typically employed when the cyst is suspected to be cancerous or is too large for laparoscopic removal. The procedure aims to excise the cyst while preserving as much healthy ovarian tissue as possible [9]. During the surgery, the surgeon makes a midline incision in the abdomen, usually extending from just above the belly button to the pubic bone. The abdominal muscles and surrounding tissues are carefully retracted to provide access to the ovaries. The cyst is excised, and the surrounding tissues are examined for any signs of malignancy. After removing the cyst, the surgeon closes the incision using sutures or staples and applies a protective bandage [10]. Potential complications associated with open cystectomy include infection, bleeding, damage to surrounding organs, and longer recovery times compared to minimally invasive techniques. The risk of complications is heightened due to the larger incision and increased manipulation of tissues. Recovery typically takes longer, with patients advised to limit physical activity for at least six to eight weeks, while full recovery may take up to 12 weeks [11]. Laparoscopic cystectomy, in contrast, is a minimally invasive surgical technique used to remove ovarian cysts through small incisions in the abdomen. This approach utilizes a laparoscope, a thin tube equipped with a camera, that allows the surgeon to visualize the surgical field without needing a large incision [12]. The laparoscopic procedure generally involves three small incisions in the lower abdomen. Carbon dioxide gas is introduced into the abdominal cavity to create space for better visibility and access. The laparoscope provides real-time images, and specialized instruments remove the cyst through one of the incisions. The gas is expelled once the cyst is removed, and the incisions are closed with dissolvable stitches [13]. While laparoscopic surgery generally presents fewer complications than open surgery, risks still exist, including infection, bleeding, and injury to surrounding organs. However, these risks are typically lower due to the procedure's minimally invasive nature. Patients often experience less postoperative pain and can return to normal activities within one to two weeks, significantly faster than recovery from open surgery [14]. An overview of the surgical techniques for open and laparoscopic cystectomy in women of reproductive age is provided in Table 1.

**Table 1:** Overview of surgical techniques for open and laparoscopic cystectomy in women of reproductive age

Surgical Technique	Indications	Procedural Details	Advantages	Disadvantages
Open cystectomy [15]	Large ovarian cysts, suspected malignancy, complex cysts	Traditional open surgery with a larger incision	Better visualization for complex cases, effective for large cysts	Longer recovery time, higher risk of adhesion formation
Laparoscopic cystectomy [16]	Benign ovarian cysts, endometriomas, simple cysts	Minimally invasive surgery using small incisions and a camera	Shorter recovery time, less postoperative pain, minimal scarring	Requires advanced surgical skills, limited visualization for large cysts
Laparoscopic ovarian cystectomy [17]	Removal of benign cysts while preserving ovarian tissue	Removal of the cyst while sparing ovarian tissue	Preserves ovarian reserve, minimally invasive	Risk of damaging ovarian tissue may require conversion to open surgery
Laparoscopic cyst aspiration [18]	Symptomatic relief in non-surgical candidates	Aspiration of cyst contents under laparoscopic guidance	Short recovery time, minimal intervention	High recurrence rate, not suitable for suspected malignancy
Robotic-assisted laparoscopy [19]	Complex benign cysts, patients with prior surgeries	Use of the robotic system for enhanced precision and flexibility	Enhanced precision and better ergonomics for the surgeon	High cost, longer operative time
Oophorectomy (open/laparoscopic) [20]	Suspected or confirmed malignancy, large benign cysts	Removal of the affected ovary can be performed open or laparoscopic	Definitive treatment for cysts reduces recurrence	Loss of ovarian function, hormonal imbalance
Laparoscopic cystectomy with ovarian suspension [21]	Prevention of adhesions in endometriosis	Cyst removal followed by suspension of the ovary to prevent adhesion	Reduced risk of adhesion formation, minimally invasive	Technically demanding, requires advanced laparoscopic skills

Fertility outcomes

Cystectomy, the surgical removal of an ovarian cyst, can significantly influence fertility through several mechanisms. A primary concern is its impact on ovarian reserve. Cystectomy can lead to a reduction in ovarian reserve, as evidenced by decreased levels of anti-Müllerian hormone (AMH) and changes in antral follicle count (AFC) [22]. While some studies indicate an initial recovery of AFC post-surgery, long-term AMH levels tend to decline significantly, which can adversely affect a woman's ability to conceive. Additionally, tissue damage during the surgical procedure may inadvertently harm surrounding ovarian tissue and its blood supply, impairing ovarian function and hormone production necessary for ovulation and conception. In cases where cystectomy is performed to treat endometriomas, the removal of these cysts may alleviate pain and improve the overall reproductive environment. This improvement can enhance fertility outcomes despite the initial reductions in ovarian reserve [23]. When comparing fertility outcomes between open and laparoscopic cystectomy, several key differences emerge. Laparoscopic cystectomy is generally associated with higher pregnancy rates, ranging from 30% to 67% within the first year post-surgery [24]. A notable study found that 64.4% of patients achieved pregnancy by the fifth year following laparoscopic cystectomy. In contrast, open cystectomy, while effective, often results in lower pregnancy rates due to greater tissue trauma and longer recovery times associated with larger incisions [24].

Moreover, laparoscopic cystectomy typically leads to shorter times to conception compared to open procedures. Many women who undergo laparoscopic surgery achieve pregnancy within the first six months post-operation, indicating a quicker recovery of ovarian function. However, it is also important to consider potential complications during pregnancy. Studies suggest that women who have undergone cystectomy may be at a higher risk for complications such as preterm labor. However, specific rates can vary based on surgical technique and individual patient factors. Additionally, there is an increased risk of ectopic pregnancies following cystectomy, particularly in cases involving significant endometriosis or previous pelvic surgeries [25]. The preservation of ovarian tissue during cystectomy is crucial in determining fertility outcomes. Laparoscopic techniques are generally more effective at preserving healthy ovarian tissue than open procedures. This preservation is vital for maintaining hormonal balance and promoting normal ovulatory cycles, which is essential for conception. Furthermore, protecting adjacent structures, such as the fallopian tubes and uterine blood supply, during surgery can enhance fertility potential by ensuring optimal conditions for fertilization and implantation [26]. Fertility outcomes associated with open and laparoscopic cystectomy in women of reproductive age are summarized in Table 2.

**Table 2:** Fertility outcomes associated with open and laparoscopic cystectomy in women of reproductive age

Fertility Outcome	Open Cystectomy	Laparoscopic Cystectomy	Comparison/Remarks
Ovarian reserve [26]	Potential reduction due to larger incision and tissue handling	Generally better preservation due to minimal ovarian tissue manipulation	Laparoscopy is preferred for preserving ovarian reserve
Anti-Müllerian hormone (AMH) levels [27]	Possible decrease post-surgery	Less reduction compared to open surgery	Laparoscopic approach shows less impact on AMH levels
Antral follicle count (AFC) [28]	Reduced AFC due to greater ovarian tissue removal	Relatively preserved AFC	Laparoscopy associated with better AFC outcomes
Pregnancy rates [29]	Slightly lower due to potential adhesion formation	Higher pregnancy rates due to lower adhesion risk	Laparoscopy is preferred for better reproductive outcomes
Time to conception [30]	Prolonged in some cases due to adhesions or reduced ovarian function	Shorter time to conception post-surgery	The laparoscopic approach facilitates a quicker return to fertility
Recurrence of cyst [31]	Lower recurrence rate but higher risk of ovarian damage	Higher recurrence rate, but preserves more ovarian tissue	The trade-off between recurrence and ovarian preservation
Adhesion formation [32]	Higher risk of pelvic adhesions	Lower risk due to minimal invasiveness	Adhesions more commonly associated with open surgery
Need for assisted reproduction techniques (ART) [33]	More likely due to compromised ovarian function	This is less likely due to better ovarian function preservation	Laparoscopic surgery reduces the need for ART in the postoperative phase
Hormonal function [33]	It may be affected depending on the extent of ovarian tissue removal	Generally preserved hormonal function	Hormonal imbalance is less likely with laparoscopic techniques
Ectopic pregnancy risk [34]	Slightly increased due to possible tubal damage	Lower risk, better tubal preservation	Laparoscopy associated with lower ectopic pregnancy risk

Factors influencing fertility outcomes

Various factors can significantly influence fertility outcomes in women of reproductive age, each playing a crucial role in determining the likelihood of conception and successful pregnancy [35]. One of the most significant determinants of fertility is the patient's age. Research indicates that female fertility begins to decline slowly in the early 30s, with a more pronounced decline occurring after age 35. By age 40, the chance of natural conception drops below 5% per month, and live birth rates decrease substantially with age [36]. For example, women aged 30 have approximately a 20% chance of conceiving each month, while this rate diminishes to approximately 5% by age 40. Additionally, older women face higher risks of miscarriage; for instance, the miscarriage rate at age 40 is about 27%, compared to 16% for those under 30. This decline in fertility with age underscores the importance of timely family planning and reproductive health awareness [37]. Underlying medical conditions, such as endometriosis and polycystic ovary syndrome (PCOS), can also adversely affect fertility. Endometriosis is known to impact ovarian function and may lead to reduced ovarian reserve and compromised oocyte quality [38]. Women with this condition often experience pain and other symptoms that complicate conception efforts. Similarly, PCOS can result in irregular ovulation, making it challenging for women to conceive naturally. These underlying conditions not only affect fertility directly but can also exacerbate the effects of aging on reproductive capabilities. Therefore, women with such conditions must seek timely medical advice and intervention to optimize their chances of conception [38].

The quality of the surgical technique employed during procedures such as cystectomy significantly influences fertility outcomes as well. Laparoscopic techniques are generally associated with better preservation of ovarian tissue compared to open cystectomy, which allows for improved fertility outcomes post-surgery [39]. The precision and skill involved in executing these surgical approaches directly affect factors such as postoperative recovery and ovarian reserve, both critical components for future conception attempts. A well-executed surgery can minimize damage to healthy ovarian tissue, thereby enhancing the potential for successful pregnancies afterward [39]. Effective postoperative care and follow-up are essential for optimizing fertility outcomes after surgical interventions. Proper management during the recovery phase can help monitor ovarian function, assess any complications that may arise post-surgery, and ensure that patients receive appropriate guidance on their reproductive health [40]. Regular follow-ups allow healthcare providers to identify any issues early and address them promptly, which can significantly enhance the likelihood of successful conception in subsequent attempts. Comprehensive care that includes emotional support and counseling can further empower women as they navigate their reproductive journeys [40]. Factors influencing fertility outcomes following open and laparoscopic cystectomy in women of reproductive age are summarized in Table 3.

**Table 3:** Factors influencing fertility outcomes following open and laparoscopic cystectomy in women of reproductive age

Factor	Description	Impact on Fertility Outcomes
Patient age [41]	Advanced maternal age (≥35) is associated with decreased ovarian reserve and reproductive potential.	Reduced ovarian reserve, lower pregnancy rates, increased risk of complications.
Type of cyst [42]	Endometriomas, dermoid cysts, functional cysts, etc.	Endometriomas may negatively impact ovarian reserve and fertility more than functional cysts.
Cyst size [43]	Larger cysts may require more extensive surgery, affecting ovarian tissue.	Increased risk of ovarian damage and decreased ovarian reserve.
Surgical technique [44]	The choice between open vs. laparoscopic, use of robotic assistance, and preservation of ovarian tissue.	Laparoscopy generally preserves ovarian function better than open surgery.
Surgeon experience [45]	Expertise in minimizing ovarian damage and preventing complications.	Experienced surgeons are more likely to preserve ovarian function and minimize adhesion formation.
Preoperative ovarian reserve [46]	Baseline ovarian reserve assessed by AMH, AFC, etc.	The low preoperative reserve may lead to poorer fertility outcomes post-surgery.
Extent of ovarian tissue removal [47]	Amount of healthy ovarian tissue removed or damaged during surgery.	Greater removal/damage is associated with reduced ovarian function.
Adhesion formation [48]	Development of pelvic adhesions post-surgery.	It can lead to infertility by affecting tubal function and ovarian mobility.
Preexisting conditions [49]	Conditions such as endometriosis, polycystic ovary syndrome (PCOS), or prior surgeries.	May worsen fertility outcomes and complicate surgery.
Postoperative recovery and complications [50]	Infection, bleeding, or prolonged recovery time.	Complications can delay conception and impact overall reproductive health.
Hormonal treatment pre/post-surgery [51]	Use of hormonal therapies like GnRH agonists to reduce cyst size or improve recovery.	It may help reduce cyst recurrence and preserve the ovarian reserve.
Recurrence of cyst [52]	Likelihood of cyst recurrence post-surgery.	Recurrence may necessitate additional surgeries, impacting ovarian reserve and fertility.
Adjuvant treatments [53]	Use of adjuvant treatments such as hormonal suppression post-surgery.	It can reduce recurrence risk but may delay the time to conception.
Surgical complications [54]	Intraoperative and postoperative complications such as bleeding or infection.	It can affect ovarian function and delay the time to conception.

Current evidence and studies

Recent studies have provided valuable insights into the comparison between open and laparoscopic techniques for cystectomy, particularly regarding ovarian endometriomas [9]. A notable study by Tsolakidis et al. conducted a prospective randomized trial that highlighted significant changes in AMH levels following cystectomy for ovarian endometriomas [55]. This research indicated that traditional cystectomy methods resulted in a considerable decrease in AMH, suggesting potential damage to ovarian reserve. The study also compared a three-step procedure involving drainage and laser treatment, finding that this approach had a lesser impact on AMH levels, thereby preserving ovarian function more effectively [55]. Another important contribution to this field is the CORAL trial, which evaluated open radical cystectomy (ORC), robotic-assisted radical cystectomy (RARC), and laparoscopic radical cystectomy (LRC). The findings revealed that ORC had higher 30-day complication rates compared to LRC, although no significant differences were observed at the 90-day mark. These results underscore the advantages of laparoscopic techniques in terms of safety and recovery [56]. Additionally, numerous comparative studies have indicated that laparoscopic approaches typically result in lower complication rates and shorter recovery periods compared to open techniques. This aligns with the findings from the CORAL trial and reinforces the growing preference for minimally invasive surgical options [13]. Meta-analyses have played a crucial role in synthesizing data from multiple studies to assess the efficacy and safety of open versus laparoscopic techniques. A comprehensive Cochrane Review analyzed five randomized controlled trials involving over 500 participants, concluding that laparoscopic approaches are associated with reduced postoperative complications compared to open methods. However, it is noted that the long-term impact on fertility remains an area that requires further investigation [57].

Other systematic reviews have emphasized the variability in outcomes based on surgical technique, highlighting the need for standardized reporting in future studies. This standardization would facilitate better comparisons across different methodologies and patient populations, ultimately leading to more reliable conclusions regarding optimal surgical practices [58]. Despite the growing body of evidence supporting laparoscopic techniques for cystectomy, several limitations persist in the current literature. One significant issue is the heterogeneity among studies regarding patient demographics, cyst characteristics, and the surgical techniques employed. This variability complicates direct comparisons between studies and may lead to conflicting conclusions [59]. Moreover, many key studies feature small sample sizes, which can limit the generalizability of their findings. For instance, while the CORAL trial provided valuable insights, it involved only 60 patients, raising concerns about statistical power and reliability. Additionally, a lack of long-term follow-up data on fertility outcomes post-surgery is prevalent in many studies. The transient nature of changes in ovarian reserve markers such as AMH may mislead interpretations about long-term fertility implications [60]. Lastly, surgeon experience can significantly influence outcomes; however, this variable is often inadequately controlled or reported in studies. Addressing these limitations is essential for advancing our understanding of the most effective surgical approaches for managing ovarian endometriomas while preserving fertility [61].

Future directions

Recent advancements in surgical techniques, particularly in minimally invasive approaches, are transforming the field of gynecological surgery. A key innovation is the increasing adoption of laparoscopic cystectomy, which offers significant advantages over traditional open surgery, such as reduced recovery time and less postoperative pain. CO_2_ fiber laser technology has further enhanced laparoscopic procedures, enabling precise tissue vaporization with minimal thermal damage, thereby preserving ovarian reserve while effectively treating endometriomas [62]. Alternatives to cystectomy, such as sclerotherapy and laser ablation, are gaining traction as they target endometrial tissue within cysts while sparing ovarian tissue, potentially reducing the risk of ovarian failure post-surgery. The development of automated feedback systems in minimally invasive procedures is also emerging, which could optimize treatment outcomes through real-time monitoring and adjustments. Additionally, innovative hybrid surgical techniques integrating imaging technologies with surgical intervention are being explored, enhancing precision and improving patient outcomes by allowing surgeons to visualize complex anatomical structures during procedures [62].

While current studies provide valuable insights into short-term outcomes following cystectomy and other interventions, there is an urgent need for comprehensive research focusing on longitudinal studies that investigate the long-term effects of various surgical techniques on fertility outcomes. This research should assess not only pregnancy rates but also the health of offspring and potential complications arising from different surgical interventions [63]. Comparative effectiveness research that contrasts laparoscopic techniques with traditional methods and newer ablative approaches will help clarify their respective impacts on ovarian function and fertility. Investigating biomarkers such as AMH levels post-surgery will enhance our understanding of how surgical techniques affect ovarian reserve over time [63]. Adopting a multidisciplinary approach is essential for optimizing gynecology and reproductive health treatment strategies. Collaboration among specialists, including gynecologists, reproductive endocrinologists, and fertility specialists, can provide comprehensive care that addresses surgical needs and fertility preservation strategies [64]. Utilizing insights from various specialties allows for tailored treatment plans considering individual patient factors such as age, health status, and reproductive goals. Multidisciplinary research efforts can lead to innovative solutions that tackle complex issues related to endometriosis and fertility, ultimately improving patient outcomes [64]. Future directions in research and clinical practice aimed at enhancing fertility outcomes following cystectomy in women of reproductive age are detailed in Table 4.

**Table 4:** Future directions in research and clinical practice for improving fertility outcomes following cystectomy in women of reproductive age ART: assisted reproduction techniques; IVF: in vitro fertilization

Future Direction	Description	Potential Impact on Fertility Outcomes
Enhanced surgical techniques [65]	Development of techniques that minimize ovarian damage and improve tissue preservation.	Better preservation of ovarian reserve and improved fertility outcomes.
Robotic-assisted surgery innovations [66]	Advancements in robotic technology for more precise and minimally invasive procedures.	Reduced risk of complications, improved ovarian preservation, and quicker recovery.
Biomarker-based preoperative assessment [67]	Biomarkers like AMH and AFC can be used to tailor surgical approaches based on individual ovarian reserve.	Personalized surgery plans to minimize ovarian damage and optimize fertility.
Fertility preservation strategies [68]	Integration of fertility preservation techniques like ovarian tissue freezing before surgery.	Allows women to preserve fertility potential, especially in high-risk cases.
Adhesion prevention techniques [48]	Research into new materials and methods to prevent postoperative adhesions.	Reduced risk of infertility due to adhesions, improved reproductive outcomes.
Impact of novel pharmacological agents [69]	Study of drugs that can protect ovarian function during and after surgery.	Potential reduction in ovarian damage and improved hormonal balance.
Comparative long-term studies [70]	Long-term studies comparing fertility outcomes of different surgical techniques over several years.	Provides evidence-based guidelines for choosing optimal surgical approaches.
Integration of ART and surgical techniques [71]	Combining surgery with assisted reproductive technologies like IVF in a single treatment plan.	Enhances the chances of conception, especially in cases with reduced ovarian reserve.
Advanced imaging for preoperative planning [72]	Use of advanced imaging modalities like 3D ultrasound and MRI to map cyst location and size.	Improved surgical planning, reduced ovarian damage, and better fertility outcomes.
Genetic and molecular research [73]	Exploration of genetic and molecular factors influencing ovarian response to surgery.	Identification of high-risk individuals tailored surgical approaches.
Patient-centered decision-making models [74]	Development of decision-making tools that consider patient preferences, risks, and fertility goals.	Improved patient satisfaction and alignment of surgical outcomes with reproductive goals.
Role of microbiome in reproductive health [75]	Investigating the impact of vaginal and gut microbiome on recovery and fertility post-surgery.	Potential for new therapies to enhance recovery and improve fertility outcomes.
Optimizing postoperative recovery protocols [76]	Research on optimal recovery protocols, including physical therapy and dietary interventions.	Faster recovery, reduced complications, and improved chances of conception.
Implementation of predictive models [77]	Use of AI and machine learning to predict surgical outcomes and fertility based on patient data.	Personalized treatment plans and improved fertility preservation.
Development of new surgical instruments [66]	Innovations in surgical tools designed specifically for fertility-preserving cystectomies.	Reduced trauma to ovarian tissue, enhanced precision, and better outcomes.

## Conclusions

The choice between open and laparoscopic cystectomy in women of reproductive age is a critical decision that significantly impacts both immediate surgical outcomes and long-term fertility. This review underscores the necessity of considering the specific indications for surgery, the unique advantages and disadvantages of each surgical technique, and the individual patient's reproductive goals. While laparoscopic cystectomy offers promising benefits such as reduced postoperative pain and shorter recovery times, it is essential for clinicians to be aware of potential complications that could influence fertility. The synthesis of current literature reveals a complex relationship between surgical technique and reproductive outcomes, emphasizing the importance of personalized surgical planning and preoperative counseling. Future research is essential to further elucidate the long-term fertility implications associated with each approach, as well as to refine surgical techniques and postoperative care strategies. Ultimately, by prioritizing both the effective management of ovarian conditions and the preservation of fertility, healthcare providers can enhance the quality of care and improve life outcomes for women navigating these challenging decisions.
